# Genome Sequence of the Alphaproteobacterium Blastochloris sulfoviridis DSM 729, Which Requires Reduced Sulfur as a Growth Supplement and Contains Bacteriochlorophyll *b*

**DOI:** 10.1128/MRA.00313-20

**Published:** 2020-04-30

**Authors:** John A. Kyndt, Dayana Montano Salama, Terry E. Meyer

**Affiliations:** a College of Science and Technology, Bellevue University, Bellevue, Nebraska, USA; b Department of Chemistry and Biochemistry, The University of Arizona, Tucson, Arizona, USA; Georgia Institute of Technology

## Abstract

The genome sequence of Blastochloris sulfoviridis is 3.85 Mb with a GC content of 68%. Its nearest relative is B. tepida (average nucleotide identity [ANI], 91.5%), followed by B. viridis (ANI, 83%). According to ANI and whole-genome-based phylogenetic analysis, the nearest relatives of *Blastochloris* are *Rhodoplanes* and *Rhodopseudomonas*, confirming the recognition of distinct genera.

## ANNOUNCEMENT

*Blastochloris* is a small genus of purple photosynthetic bacteria, distinguished by using bacteriochlorophyll *b* as its photosynthetic pigment and absorbing light near 1,000 nm as opposed to the bacteriochlorophyll *a*-containing purple bacteria, which absorb in the region of 860 nm (1). Currently, there are only four species recognized, Blastochloris viridis (2), B. sulfoviridis (3), B. gulmargensis (4), and B. tepida (5, 6). They were originally placed in the genus *Rhodopseudomonas* but were transferred to the new genus, *Blastochloris*, by Hiraishi (7). In addition to its unusual bacteriochlorophyll, *B. sulfoviridis* uses thiosulfate as a growth substrate. In fact, a reduced sulfur source is essential for growth because it lacks assimilatory sulfate reduction. *B. viridis* was the first photosynthetic species to have the structure of its reaction center solved (8), for which a Nobel Prize was awarded. The genome sequences for *B. viridis* and *B. tepida* had previously been determined (6, 9).

Blastochloris sulfoviridis DSM 729 was originally isolated from a sulfur spring (in the former USSR) (3). Cells were grown and genomic DNA was prepared by DSMZ (Deutsche Sammlung von Mikroorganismen und Zellkulturen, GmbH). Qubit and NanoDrop DNA analysis showed an *A*_260_/*A*_280_ ratio of 1.80. The sequencing library was prepared using the Illumina Nextera DNA Flex library prep kit. The genome was sequenced with an Illumina MiniSeq using 500 μl of a 1.8 pM library. Paired-end (2 × 150-bp) sequencing generated 2,355,376 reads and 183.3 Mbp (35× coverage). Quality control of the reads was performed using FastQC within BaseSpace version 1.0.0 (Illumina), using a k-mer size of 5 and contamination filtering. We assembled the genome *de novo* using SPAdes version 3.10.0 (10) through PATRIC (11). This assembly yielded 91 contigs (>300 bp), the largest being 221,883 bp with an *N*_50_ value of 108,240 bp. The genome was 3,845,334 bp long, and the GC content was 68.1%. The genome was annotated using RASTtk (12) within PATRIC (11). This showed our strain to have 3,759 coding sequences and 46 tRNAs.

**FIG 1:**
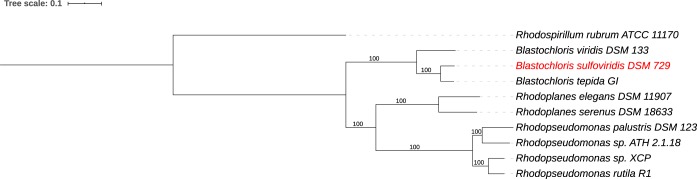
Phylogenetic tree of whole-genome comparison of Blastochloris sulfoviridis to its closest relatives. The phylogenetic tree was generated using the codon tree method within PATRIC (10), which used PGFams as homology groups and analyzed 634 aligned proteins and coding DNA from single-copy genes using RAxML (14, 15). The support values for the phylogenetic tree were generated using 100 rounds of the “Rapid bootstrapping” option of RAxML (14). Rhodospirillum rubrum ATCC 11170 was added as an outgroup. iTOL was used for the tree visualization (17). The tree scale is defined as the mean number of substitutions per site.

A JSpecies comparison (13) of average percentage nucleotide identity (ANIb) between the *B. sulfoviridis* genome and its nearest relatives yielded 91.5% identity with the *B. tepida* genome and 83.0% with *B. viridis* (Table 1). The ANI values with the *Rhodopseudomonas* and *Rhodoplanes* genomes were below 75%. The ANI numbers for *B. sulfoviridis* are below the 95% cutoff for the genomic definition of a species (13). Phylogenetic analysis of the *B. sulfoviridis* genome using RAxML within PATRIC (14, 15) showed *B. tepida* as the closest relative, followed by *B. viridis*, and clearly different from the genera *Rhodopseudomonas* and *Rhodoplanes* (Fig. 1).

**TABLE 1:** ANIb comparisons of Blastochloris sulfoviridis and its closest relatives

Organism	% avg nucleotide identity with:			
	*B. sulfoviridis* DSM 729^T^	*B. tepida* DSM 106918^T^	*B. viridis* DSM 133^T^	*Rhodoplanes elegans* DSM 11907
*B. tepida* DSM 106918^T^	91.5			
*B. viridis* DSM 133^T^	83.0	82.9		
*Rhodoplanes elegans* DSM 11907	73.4	73.6	72.9	
*Rhodopseudomonas palustris* DSM 123^T^	71.4	71.6	71.7	72.0

Both *Blastochloris* and *Rhodoplanes* species presumably utilize a small cytochrome *c*_2_ as the electron donor to the photosynthetic reaction center, but *Rhodopseudomonas* species use an extra-large cytochrome *c*_2_ and are lacking the *pufC* cytochrome subunit of the reaction center (the extra-large *c*_2_ has 3 and 14 residue insertions compared with small *c*_2_; 16). The *B. sulfoviridis* and *B. tepida* genomes contain the sox operon for dissimilatory thiosulfate oxidation, but *B. viridis* does not. All of the *Blastochloris* genomes apparently have two sets of genes for assimilatory nitrogen fixation, the normal Mo/Fe (Nif) and the less common Fe/Fe (Anf) enzyme, and they also have the MtrAB/high-potential iron-sulfur protein (HiPIP) operon for dissimilatory Fe(II) oxidation.

### Data availability.

This whole-genome shotgun project has been deposited at DDBJ/ENA/GenBank under the accession number VWPL00000000. The version described in this paper is version VWPL01000000. The raw sequencing reads have been submitted to SRA, and the corresponding accession number is SRR11362080.
